# Supplementary material for: Determinants of sustainable solid waste management in Jimma City, Southwest Ethiopia
Source: PLoS One. 2025 Sep 26;20(9):e0333170. doi: 10.1371/journal.pone.0333170 (PMC12469378; doi:10.1371/journal.pone.0333170)
Supplement: S1 File — (DOCX) [file pone.0333170.s001.docx]

**Solid Waste Management Sustainability Level Practices Classification**

The SW management practices and the sustainability level of the study participants were measured by using this checklist.

| **Solid Waste Management Sustainability Category** | **Description** | **Examples of Practices** | **Environmental Impact and Resource Efficiency** |
| --- | --- | --- | --- |
| Unsustainable | - Practices that disregard waste hierarchy, cause environmental harm, and are inefficient in resource use. | - Open dumping without segregation - Uncontrolled burning/incineration - Landfilling without segregation | - High pollution and health risks - Low resource recovery - Negative ecological effects |
| Moderate | - Practices that partially follow waste hierarchy principles but lack comprehensive infrastructure and systematic implementation. | - Partial segregation of solid waste - Informal recycling or composting - Irregular collection and disposal | - Moderate reduction in pollution - Some resource recovery - Limited environmental benefits |
| Sustainable | - Practices are fully aligned with closed-loop, circular economy principles, emphasizing waste reduction and resource recovery. | - Source segregation at the household or community level - Reuse of materials - Energy recovery from waste - Organized community recycling programs | - Low environmental impact - High resource efficiency and recovery - Supports circular economy |

**Sustainable solid waste management practices analysis results**

| **Sustainable SWM** | **Freq.** | **Percent** | **Cum.** |
| --- | --- | --- | --- |
| Unsustainable SWM | 231 | 28.17 | 28.17 |
| Moderate | 518 | 63.17 | 91.34 |
| Sustainable SWM | 71 | 8.66 | 100 |
| **Overall** | **820** | **100** |  |
