# Supplementary material for: Determinants of sustainable solid waste management in Jimma City, Southwest Ethiopia
Source: PLoS One. 2025 Sep 26;20(9):e0333170. doi: 10.1371/journal.pone.0333170 (PMC12469378; doi:10.1371/journal.pone.0333170)
Supplement: S2 File — (DOCX) [file pone.0333170.s002.docx]

**Full information on the data analysis model output**

**Ordinal Logistic Regression Estimates**

| **Sustainable SWM** | **Coefficient** | **Robust SE** | **z** | **p>z** | **[95% Conf. Interval]** | |
| --- | --- | --- | --- | --- | --- | --- |
| **HH_Head** |  |  |  |  |  |  |
| Male | 0.3515233 | 0.1859035 | 1.89 | 0.059 | -0.01284 | 0.715887 |
| **Education** |  |  |  |  |  |  |
| Primary Education | 0.7007428 | 0.254244 | 2.76 | 0.006 | 0.202434 | 1.199052 |
| Secondary Education | 0.8702783 | 0.2688991 | 3.24 | 0.001 | 0.343246 | 1.397311 |
| Tertiary Qualifications | 0.4149166 | 0.2360926 | 1.76 | 0.079 | -0.04782 | 0.87765 |
| **Income Category** |  |  |  |  |  |  |
| Middle Income | -0.2683422 | 0.2478073 | -1.08 | 0.279 | -0.75404 | 0.217351 |
| Higher Income | 0.5681308 | 0.2467676 | 2.3 | 0.021 | 0.084475 | 1.051786 |
| **Dwelling Ownership** |  |  |  |  |  |  |
| Rented | 0.092356 | 0.1853401 | 0.5 | 0.618 | -0.2709 | 0.455616 |
| **Resid. Duration** | 0.0183566 | 0.0067853 | 2.71 | 0.007 | 0.005058 | 0.031656 |
| **Waste Gen. kg/cap/day** | 0.1471211 | 0.0492707 | 2.99 | 0.003 | 0.050552 | 0.24369 |
| **Attitude** |  |  |  |  |  |  |
| Moderate | 0.3981208 | 0.2544346 | 1.56 | 0.118 | -0.10056 | 0.896804 |
| Favourable | 0.7235718 | 0.2842913 | 2.55 | 0.011 | 0.166371 | 1.280773 |
| **Kebele** |  |  |  |  |  |  |
| Hirmata_Mentina | 1.466456 | 0.4156449 | 3.53 | 0.000 | 0.651807 | 2.281105 |
| Mendera_Kochi | 1.443036 | 0.4055182 | 3.56 | 0.000 | 0.648236 | 2.237837 |
| Ginjo_Guduru | 1.993968 | 0.4300044 | 4.64 | 0.000 | 1.151175 | 2.836761 |
| Bacho_Bore | 0.9142446 | 0.3850876 | 2.37 | 0.018 | 0.159487 | 1.669002 |
| Bore | 1.733409 | 0.4381376 | 3.96 | 0.000 | 0.874675 | 2.592143 |
| /cut1 | 2.277103 | 0.5181841 |  |  | 1.261481 | 3.292725 |
| /cut2 | 6.005957 | 0.5606349 |  |  | 4.907132 | 7.104781 |

**Post-regression Estimates of Average Marginal Effects of Predictors**

|  | **dy/dx** | **SE** | **Z** | **p>z** | **[95% Conf. Interval]** | |
| --- | --- | --- | --- | --- | --- | --- |
| 1.HH_Head |  |  |  |  |  |  |
| 2.HH_Head |  |  |  |  |  |  |
| _predict_SSWM | 0.0242 | 0.012012 | 2.01 | 0.044 | 0.000625 | 0.047712 |
| 0.Educ |  |  |  |  |  |  |
| 1.Educ |  |  |  |  |  |  |
| _predict_SSWM | 0.0466 | 0.018835 | 2.47 | 0.013 | 0.009679 | 0.083509 |
| 2.Educ |  |  |  |  |  |  |
| _predict_SSWM | 0.0621 | 0.021522 | 2.88 | 0.004 | 0.019875 | 0.10424 |
| 3.Educ |  |  |  |  |  |  |
| _predict_SSWM | 0.0245 | 0.013036 | 1.88 | 0.06 | -0.00107 | 0.050033 |
| 1.Income_Ctgry |  |  |  |  |  |  |
| 2.Income_Ctgry |  |  |  |  |  |  |
| _predict_SSWM | -0.0138 | 0.013146 | -1.05 | 0.296 | -0.03952 | 0.012014 |
| 3.Income_Ctgry |  |  |  |  |  |  |
| _predict_SSWM | 0.0411 | 0.016289 | 2.52 | 0.012 | 0.009159 | 0.073012 |
| 1.Dwelling_Own. |  |  |  |  |  |  |
| 2.Dwelling_Own. |  |  |  |  |  |  |
| _predict_SSWM | 0.0068 | 0.013633 | 0.5 | 0.617 | -0.0199 | 0.033536 |
| ResDurtn |  |  |  |  |  |  |
| _predict_SSWM | 0.0014 | 0.000507 | 2.67 | 0.008 | 0.000359 | 0.002344 |
| HH_Wastekg/cap/day |  |  |  |  |  |  |
| _predict_SSWM | 0.0108 | 0.00369 | 2.94 | 0.003 | 0.003599 | 0.018064 |
| 1.Attitude |  |  |  |  |  |  |
| 2.Attitude |  |  |  |  |  |  |
| _predict_SSWM | 0.0238 | 0.013721 | 1.73 | 0.083 | -0.00312 | 0.050662 |
| 3.Attitude |  |  |  |  |  |  |
| _predict_SSWM | 0.0495 | 0.018952 | 2.61 | 0.009 | 0.012351 | 0.086643 |
| 1.Kebele |  |  |  |  |  |  |
| 2.Kebele |  |  |  |  |  |  |
| _predict_SSWM | 0.0622 | 0.019753 | 3.15 | 0.002 | 0.023443 | 0.100874 |
| 3.Kebele |  |  |  |  |  |  |
| _predict_SSWM | 0.0604 | 0.017665 | 3.42 | 0.001 | 0.025826 | 0.095072 |
| 4.Kebele |  |  |  |  |  |  |
| _predict_SSWM | 0.1098 | 0.026918 | 4.08 | 0.000 | 0.057031 | 0.162545 |
| 5.Kebele |  |  |  |  |  |  |
| _predict_SSWM | 0.0293 | 0.010701 | 2.74 | 0.006 | 0.008369 | 0.050316 |
| 6.Kebele |  |  |  |  |  |  |
| _predict_SSWM | 0.0840 | 0.019676 | 4.27 | 0.000 | 0.04541 | 0.122536 |

Note: dy/dx for factor levels is the discrete change from the base level using Delta-method.
