# Supplementary material for: Determinants of sustainable solid waste management in Jimma City, Southwest Ethiopia
Source: PLoS One. 2025 Sep 26;20(9):e0333170. doi: 10.1371/journal.pone.0333170 (PMC12469378; doi:10.1371/journal.pone.0333170)
Supplement: Attachment — Submitted filename: Response to Reviewers Revised.docx [file pone.0333170.s004.docx]

**Authors’ responses to the editor’s and reviewers' questions and comments**

We appreciate the editor and the reviewers for their rational and constructive comments and thorough revision of our paper. Your valuable and insightful comments led to possible improvements in the current version. We have addressed all comments and considered the suggestions for the manuscripts. We hope the manuscript, after careful revisions, meets your high standards. The authors welcome further comments, if any. All modifications in the manuscript have been tracked in red.

**Response to editor comments on Journal requirements:**

**Comment 1:** If the reviewer comments include a recommendation to cite specific previously published works, please review and evaluate these publications to determine whether they are relevant and should be cited.

**Response:** Thank you for your reminder regarding the citation of previously published works. In this case, neither the editor nor the reviewers suggested any additional references to be cited. Therefore, no changes were made to the citations or reference list in the revised manuscript.

**Comment 2:** Please review your reference list to ensure that it is complete and correct. If you have cited papers that have been retracted, please include the rationale for doing so in the manuscript text, or remove these references and replace them with relevant current references.

**Response:** Thank you for your critical reminder regarding the reference check. We have carefully reviewed the reference list in the manuscript and confirm that all citations are correct and complete. To the best of our knowledge, none of the cited works has been retracted. Therefore, no changes have been made to the reference list.

**REVIEWER 1**
**Comments to the Author**:

**General comments:** The manuscript has been significantly improved since the first draft.

**Response:** We sincerely appreciate your positive feedback and are grateful for your recognition of the improvements made to the manuscript.

**Comment 1:** Lines 90-92: Please state which year(s) these figures refer to. As waste arisings are increasing so quickly, it is useful to know how recent this data is.

**Response:** Thank you for your critical comments. We have incorporated the year of the published data into the citation in the revised manuscript as suggested.

**Comment 2:** Line 105: By “evergreen,” do you mean “agricultural”? Replace the full stop at the end of this short sentence with a comma to incorporate it into the first part of the next sentence, making the next sentence shorter.

**Response:** Thank you for your comments and suggestions. We have replaced the word “evergreen” with “year-round agricultural activities” and merged the short sentence with the first part of the following sentence, thereby shortening it as per your suggestion. The revised sentences have been included in the updated manuscript.

**Comment 3:** Line 107: No exception to what? Suggest removing this and starting the sentence with “In Jimma City…”

**Response:** Thank you for your constructive comment and suggestion. We have removed the previous words and revised the sentence to start with “In Jimma City…” in the revised manuscript as recommended.

**Comment 4:** Lines 323 and 333: The numbers are not visible when referring to the Figures.
**Response:** Thank you for your constructive comment. We have adjusted the font size and ensured that both figures on pages 323 and 333 are visible in the revised manuscript.

**Comment 5:** In Table 4, the < symbols are not visible.

**Response:** Thank you for your careful observation and comment regarding Table 4. The missing “<” symbols have now been inserted into the table in the revised manuscript.
